# Non-tuberculous mycobacteria isolates from patients with chronic pulmonary disease and no epidemiological relationship show sequence clusters through whole-genome sequencing

**DOI:** 10.3389/fmicb.2025.1549030

**Published:** 2025-03-11

**Authors:** Marc Rubio, Mariana Fernandez-Pittol, Sara Batista, Diego Martínez, Lorena San Nicolas, Elena Portell-Buj, Maria Antònia Busquets, Joan Estelrich, Julian Gonzalez-Martin, Ferran Navarro, Griselda Tudó, Montserrat Garrigó

**Affiliations:** ^1^Servei de Microbiologia, Fundació de Gestió de l'Hospital de la Santa Creu i Sant Pau, Barcelona, Spain; ^2^Institut d'Investigació Biomèdica Sant Pau (IIB Sant Pau), Barcelona, Spain; ^3^Departament de Fonaments Clínics, Facultat de Medicina i Ciències de la Salut, Universitat de Barcelona, Barcelona, Spain; ^4^ISGlobal Barcelona, Institute for Global Health, Barcelona, Spain; ^5^Servei de Microbiologia, CDB, Hospital Clínic de Barcelona, Barcelona, Spain; ^6^Institut d’Investigacions Biomèdiques August Pi i Sunyer (IDIBAPS) Rosselló, Barcelona, Spain; ^7^Department de Farmàcia, Tecnologia Farmacèutica i Físicoquímica, Facultat de Farmàcia i Ciències de l'Alimentació, Universitat de Barcelona, Barcelona, Spain; ^8^Institut de Nanociència i Nanotecnologia, IN2UB, Facultat de Química, Barcelona, Spain; ^9^CIBER of Infectiuos Diseases (CIBERINFEC), Instituto de Salud Carlos III, Madrid, Spain; ^10^Department of Genetics and Microbiology, Universitat Autonoma de Barcelona, Cerdanyola del Valles, Barcelona, Spain

**Keywords:** non tuberculous mycobacteria, whole genome sequencing, molecular epidemiology, cluster analysis, bioinformatics

## Abstract

**Objectives:**

This study aimed to investigate the genomic epidemiology of slow-growing mycobacteria (SGM) isolates from patients with bronchiectasis through whole-genome sequencing (WGS) and assess various bioinformatic tools to establish relationships between the isolates.

**Methods:**

A total of 46 SGM isolates from 37 patients with underlying chronic pulmonary disease, previously identified as *Mycobacterium avium*, *Mycobacterium intracellulare*, or *Mycobacterium chimaera* through polymerase chain reaction, were analyzed using WGS and three different clustering methods, namely rPinecone, Split K-mer analysis (SKA), and custom single nucleotide variant threshold calculation.

**Results:**

The three analyses revealed one cluster of *M. intracellulare* subsp. *intracellulare* isolates and one cluster of *M. intracellulare* subsp. *chimaera* isolates from different patients. The analyses did not indicate any clusters formed by *M. avium* subsp*. avium* isolates from different patients.

**Conclusion:**

*M. intracellulare* subsp. *chimaera* and *M. intracellulare* subsp. *intracellulare* form clusters of very closely related isolates from patients with no epidemiological relationship. This absence of an epidemiological relationship indicated that the infections were likely acquired from common sources rather than through direct transmission between patients. The use of three methodologies is an adequate strategy for an in-depth study of the relationship between isolates of very closely related species and subspecies.

## 1 Introduction

*Mycobacterium avium* and *Mycobacterium intracellulare* are two closely related species of non-tuberculous mycobacteria (NTM) that belong to the *M. avium* complex (MAC) (Schoch et al., 2020; Daley et al., 2020). *M. avium* is formed by *M. avium* subsp. *avium*, *M. avium* subsp. *paratuberculosis,* and *M. avium* subsp. *silvaticum*. *M. intracellulare* is formed by *M. intracellulare* subsp. *intracellulare, M. intracellulare* subsp. c*himaera* and *M. intracellulare* subsp. *yongonense* (list of prokaryotic names with standing in nomenclature is available at https://lpsn.dsmz.de/). *M. avium* and *M. intracellulare* are both included in the slow-growing mycobacteria (SGM) group NTM and can lead to infections in immunosuppressed patients, being especially frequent in patients with chronic pulmonary diseases or patients with HIV (Hoefsloot et al., 2013; Johnson and Odell, 2014; Zweijpfenning et al., 2018).

The origin of NTM infections is environmental. In nature, NTM are found not only in soil and water but also in environments that are very close to human life, such as shower heads, tap water, household fomites, and even hospital water systems. MAC are the most common species and are broadly distributed (Hoefsloot et al., 2013; Falkinham, 2015; Winburn and Sharman, 2023). Although human exposure to NTM is common, and colonization of the airways can occur, the development of infection is generally associated with patients who have impaired lung defenses, such as those with bronchiectasis and cystic fibrosis. In addition, NTM can develop disseminated infections in immunocompromised patients (Johnson and Odell, 2014; Winburn & Sharman, 2023). Patient-to-patient transmission of NTM infection is unlikely to occur and is not considered the main source of NTM transmission. Although some studies have identified an epidemiological relationship between isolates from patients infected with *M. abscessus* subsp. *massiliense* in a cohort of cystic fibrosis patients (Bryant et al., 2016). However, the prevailing view is that transmission between humans occurs through the contamination of fomites by respiratory secretions (Bryant et al., 2016).

The pulmonary infections caused by NTM in patients with chronic lung diseases are generally less aggressive than tuberculosis, but they are considerably more complex. The evolution of the infection is often progressive and treatments may fail due to the intrinsic antimicrobial resistance found in NTM.

In this study, whole-genome sequence analysis was performed to study the phylogenetic relationship of SGM causing lung infections in patients with chronic pulmonary disease as a predisposing factor. Therefore, two objectives were established: first, to detect potential transmission events among 46 SGM isolates obtained from 35 patients with a history of chronic lung disease treated at the same hospital (Hospital Clinic of Barcelona); and second, to evaluate various bioinformatic approaches for studying the epidemiological relationship between isolates.

## 2 Methodology

### 2.1 Patients and sample collection

We collected 46 SGM isolates from 37 patients with bronchiectasis, a chronic pulmonary disease (Supplementary material). The strains were previously identified as *M. avium*, *M. intracellulare*, or *Mycobacterium chimaera* by matrix-assisted laser desorption/ionization–time of flight (Buckwalter et al., 2016) and polymerase chain reaction [including sequencing of *16S* and *rpoB* genes (Adékambi and Drancourt, 2004; Adékambi et al., 2006)].

### 2.2 Whole-genome sequencing

The 46 isolates were analyzed using whole-genome sequencing (WGS). Genomic DNA was extracted from all the isolates, as described previously (Doyle et al., 2020), with some adjustments: DNA was extracted using the Qiagen EZ1 DNA Tissue Extraction Kit (Qiagen, Madrid, Spain) after a previous step of bead-beating. Total DNA was determined using Qubit (Thermofisher) and DNA quality was determined using a NanoDrop spectrophotometer (Thermofisher). Genomic DNA extracts were sent to NovoGene (NovoGene Europe INC, Cambridge, UK) for WGS. WGS was performed in Illumina NovaSeq with 2×150 paired-end reads.

### 2.3 Bioinformatics pipeline

From raw reads to species identification or genomic epidemiology analysis, different processes detailed in the next sections were performed. A synthesis flowchart with the key points of these processes is included. All the scripts used in this study are available at https://github.com/hsp-microbiology/wgs_mycobacterium_pipeline.

### 2.4 *De novo* assembly and species identification

Sequenced reads of all the samples were trimmed using fastp (available at https://github.com/OpenGene/fastp); *de novo* assembly using Unicycler v0.5.0 package (available at https://github.com/rrwick/Unicycler), a wrapper program based on SPAdes assembler was performed (Wick et al., 2017). The first screening for species identification was carried out by submitting assembled fasta files to the PubMLST Identify species database (available at https://pubmlst.org/bigsdb?db=pubmlst_rmlst_seqdef_kiosk). Species identification was also performed by calculating the average nucleotide identity (ANI) between the assembled genomes and the reference strain genomes using orthoANI (Lee et al., 2016). ANI values ≥95% indicated that the isolates and type strain genomes were the same species, and ANI values ≥98% indicated that they were the same subspecies.

Reference strain genomes used for comparison were obtained from the National Center for Biotechnology Information (NCBI) (available at https://www.ncbi.nlm.nih.gov/) and are shown in Supplementary material.

### 2.5 Genome annotation and SGM distribution

*De novo* assembled genomes and publicly available reference genomes (Supplementary Table S2) were annotated using Prokka v1.14.6 (available at https://github.com/tseemann/prokka) (Prokka, 2014). General Feature Format (gff) files containing protein information and annotation from the first isolate of every patient were used as Roary input (available at https://github.com/sanger-pathogens/Roary) (Page et al., 2015). Roary was executed for all NTM strains with a minimum identity percentage of 90% for blastp, and genes had to be present in 99% of isolates to be called a core gene. Core-gene alignment was used to infer a maximum likelihood (ML) tree in RaxML v8.2.12 (Stamatakis, 2014). The phylogenetic tree was visualized and annotated using Figtree (available at https://github.com/rambaut/figtree). Publicly available genomes were also used at this point (Supplementary material).

### 2.6 Variant calling and phylogenetic analysis

Trimmed reads were mapped to their closest reference strain genome using Snippy v4.6.0 (Seemann, 2014). Single nucleotide variants (SNVs) were called against the reference genome using freebayes v1.3.2 incorporated in Snippy (Garrison and Marth, 2012). Potential regions with high recombination events were identified and removed using Gubbins v3.2.1. Variants were filtered in a 10,000 bp sliding window (Croucher et al., 2015). A maximum likelihood tree was inferred from every species cluster using RAxML v8.2.12 (Stamatakis, 2014) and a General Time Reversible model with 1,000 bootstrap sampling.

Distinct subtrees were inferred for each species: *M. avium* subsp. *avium*, *M. intracellulare* subsp. *chimaera* and *M. intracellulare* subsp. *intracellulare*. All the isolates in each subtree were mapped against the closest reference genome available. Strains that were not identified at the subspecies level or were mixtures of different species using our pipeline were excluded from this analysis.

### 2.7 Sequence cluster analysis

Sequence clusters for inferring molecular epidemiology were generated using three different approaches. First, an SNV threshold was obtained from the maximum diversity within the same patient samples and was applied to hierarchical clustering based on the pairwise SNV matrix. The upper boundary limit was taken as the SNV threshold to cluster the isolates into ‘clones’ if they were below this threshold or ‘not clones’ if they were above this threshold (Doyle et al., 2020).

Second, rPinecone (Wailan et al., 2019) was executed in the ML SNV-generated tree using an SNV threshold consistent with that established in the previous step. Assembled genomes were processed through the pubMLST typing database pipeline for *Mycobacteria* spp. to obtain the multilocus sequence typing (MLST) profile for our strains^1^. Different R packages were employed to annotate the trees: ‘seqinr’, ‘NMF’, ‘reshape2’, ‘RcolorBrewer’, ‘ggtree’, ‘ape’, ‘phangorn’, ‘tidyverse’, ‘ggh4x’, ‘adegenet’, and ‘ggstance’, available from the Comprehensive R Archive Network (CRAN) repository, BiocManager, and GitHub.

Third, to support our findings and methodology, (Split K-mer Analysis (SKA) is available at https://github.com/simonrharris/SKA) was used. SKA is a toolkit for prokaryotic DNA sequence analysis suited for surveillance or outbreak investigation (Harris, 2018). We used default settings: an SNP cutoff of 20 and the proportion of SKA of 0.9.

All the clinical isolates were analyzed using these three methods.

## 3 Results

### 3.1 Slow-growing mycobacteria species identification

Initially, WGS data of 46 clinical isolates obtained from 35 patients were obtained; there were 15/46 *M. avium* subsp. *avium* isolates from 12 patients; 26/46 *M. intracellulare* isolates from 20 patients distributed into 9/26 *M. intracellulare* subsp. *chimaera* isolates from 8 patients, 13/26 *M. intracellulare* subsp. *intracellulare* isolates from 11 patients, and 4/26 *M. intracellulare* sp. from 3 patients; 1/46 *M. paraintracellulare* from 1 patient; 3/46 contaminations from 3 patients; and 1/46 mixed NTM species from 1 patient. These four samples from three patients were excluded from downstream analyses because *de novo* assembly, mapping to reference, or pubMLST species identification revealed mixed NTM species or contamination (details in Supplementary material).

**Figure 1:**
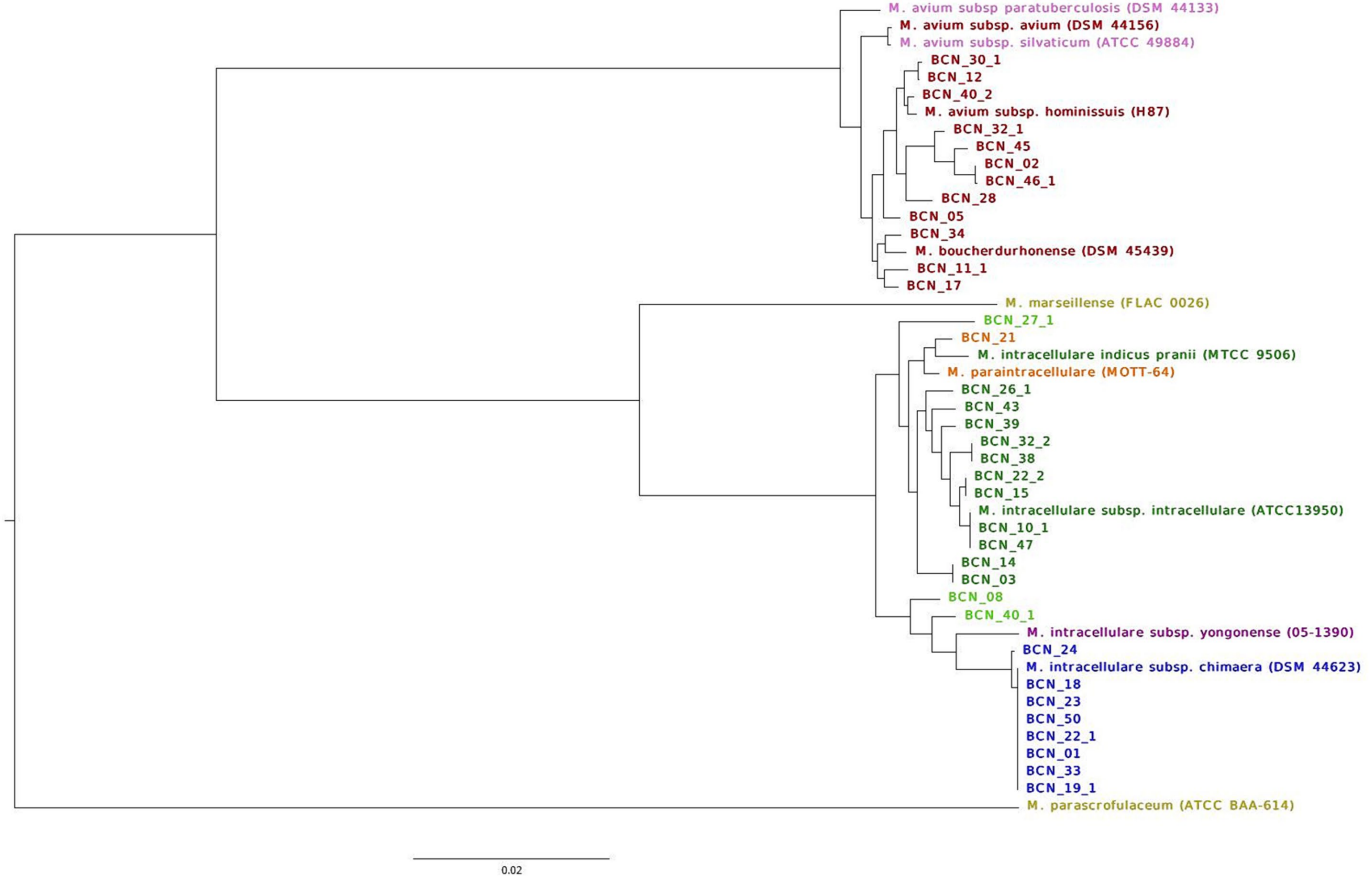
Classification of NTM isolates in this study into species and subspecies using well-annotated reference type genomes. The figures show the maximum likelihood of the SNV tree inferred from core-genome alignment for all SGM first isolates and type strain genomes. SNVs were identified from core-genome Roary alignment, based on blastp 90% identity. Samples are colored based on WGS strain identification. *M. avium* subsp. *avium* strains in red; *M avium* subsp. *paratuberculosis,* and *M. avium* subsp. *silvaticum* reference strains in pink; *M. marseillense* and *M. parascrofulaceum* reference genomes used as outgroups in yellow; *M. intracellulare* subsp. *intracellulare* genomes in dark green; *M. paraintracellulare* in orange; *M. intracellulare* subsp. *yongonense* reference genome in purple; *M. intracellulare* subsp. *chimaera* genomes in blue; and *M. intracellulare* sp. strains in light green. The scale bar indicates the average number of substitutions per site (23,640 SNVs).

The ANI percentage identity (Supplementary material) correctly classified 38 out of 42 (90%) isolate genomes. Two isolates from two patients (BCN_08 and BCN_40_1) were classified as *M. intracellulare* sp. The ANI percentage identity, pubMLST, and Roary core-gene SNV analysis of these two strains did not cluster them with any subspecies. They were classified in the main *M. intracellulare* branch between *M. intracellulare* subsp. *yongonense* branch and *M. intracellulare* subsp. *intracellulare* branch. Two isolates from the same patient (BCN_27_1 and BCN_27_2) showed a 94% genome correlation with *M. intracellulare* and a 6% correlation with *M. paraintracellulare* and were also clustered inside the *M. intracellulare* branch (Roary SNV tree, Figure 1).

Roary analysis with a percentage of identity of 90% in blastp revealed 1,130 core genes (present in >99% of genomes), 919 softcore genes (present in 95–99% of genomes), 6,368 shell genes (found in 15–95% of the genomes), and 14,093 cloud genes (found in <15% of the genomes) from a total of 22,510 genes present in 35 first isolates from 32 patients (three patients with different SGM isolated on separate dates) and 12 reference genome sequences (accession numbers available in Supplementary Table S1). The maximum likelihood tree derived from the core-genome alignment exhibits a distribution that supports SGM identification. The tree shown in Figure 1 displays two well-defined branches supported by *M. parascrofulaceum* as an outgroup for MAC genomes and *M. marseillense* as an outgroup for *M. intracellulare* genomes.

### 3.2 Sequence cluster analysis

**Figure 2:**
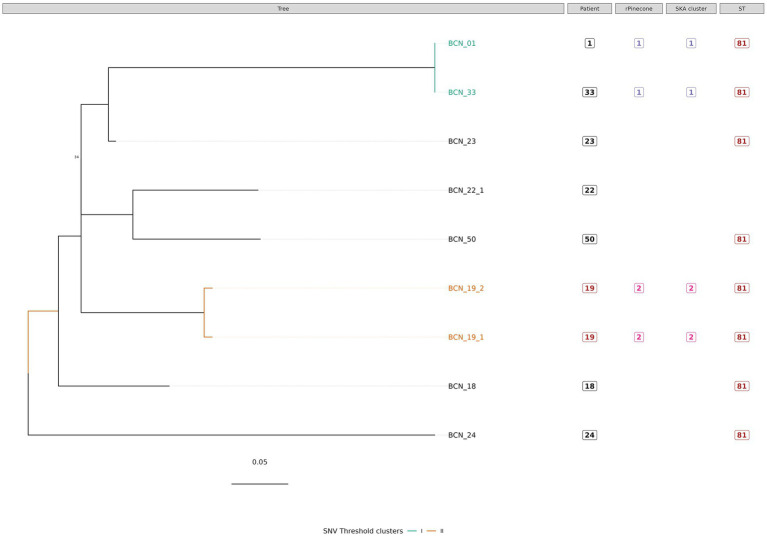
Phylogenetic tree of *M. intracellulare s*ubsp. *chimaera* isolates with sequence clustering data, patient information, and Sequence Type (ST) information annotated. This figure shows the maximum likelihood SNV tree for all *M. intracellulare* subsp. *chimaera* isolates. The SNVs were identified from mapping reads to *M. intracellulares* subsp. *chimaera* reference genome DSM 44,623. Highlighted samples correspond to the sequence cluster calculated using the SNV threshold from the upper boundary of the SNV pairwise distance within patient diversity. The scale bar shows the number of SNVs per site (6.9 SNVs) and node bootstrap scores are shown when below 75 (with 1,000 bootstrap sampling). The tree figure was annotated with additional information in columns: Patient, from which the strain was isolated; rPinecone, cluster number generated from rPinecone R analysis; SKA cluster, cluster number generated from SKA analysis; and ST, sequence type profile number obtained from the pubMLST *Mycobacteria* spp. scheme.

**Figure 3:**
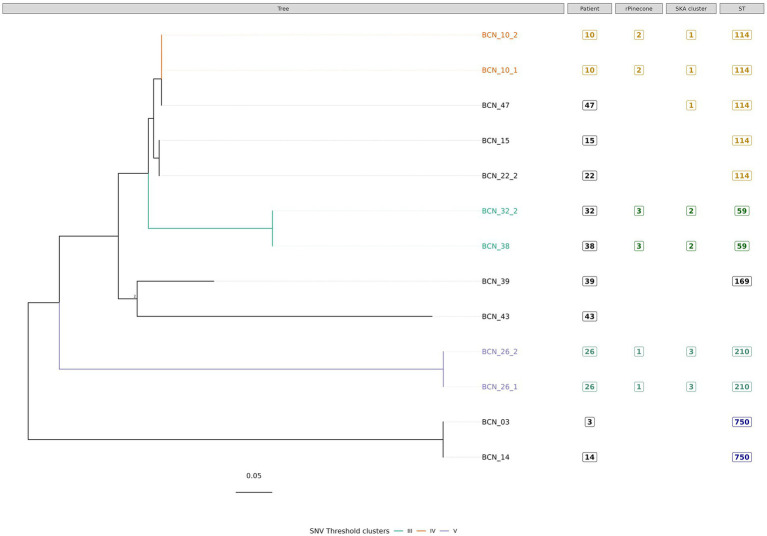
Phylogenetic tree of *M. intracellulare s*ubsp. *intracellulare* isolates with sequence clustering data, patient information, and Sequence Type (ST) information annotated. This figure illustrates the maximum likelihood SNV tree for all *M. intracellulare* subsp. *intracellulare* isolates. SNVs were identified from mapping reads to *M. intracellulare* subsp. *chimaera* reference genome ATCC 13,950 (accession number: NC_016946.1). Highlighted samples correspond to the sequence cluster calculated using the SNV threshold from the upper boundary of SNV pairwise distance within patient diversity. The scale bar shows the number of SNVs per site (1,049.95 SNVs), and node bootstrap scores are shown when below 75 (with 1,000 bootstrap sampling). The tree figure was annotated with additional information in columns: Patient, from which the strain was isolated; rPinecone, cluster number generated from rPinecone R analysis; SKA cluster, cluster number generated from SKA analysis; and ST, the sequence type profile number obtained from the pubMLST *Mycobacteria* spp. scheme.

**Figure 4:**
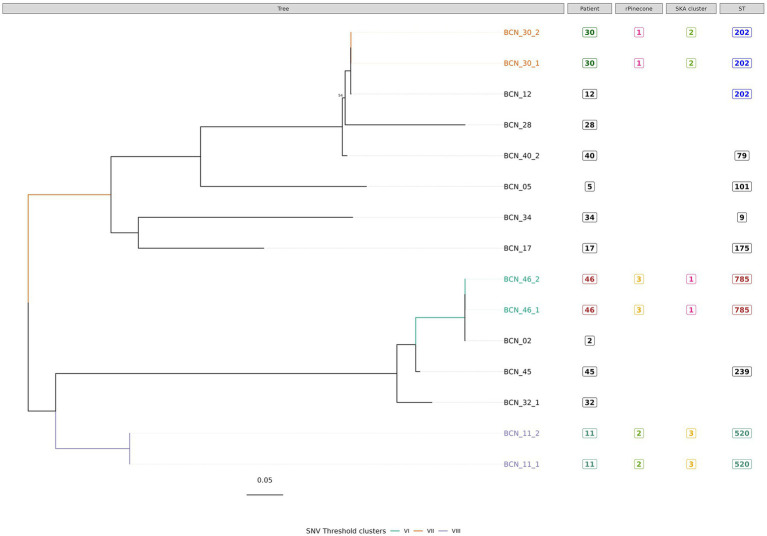
Phylogenetic tree of *M. avium s*ubsp. *avium* isolates with sequence clustering data, patient information and Sequence Type (ST) information annotated. This figure illustrates the maximum likelihood SNV tree for all *M. avium* subsp. *avium* isolates. SNVs were identified from mapping reads to *M. avium* subsp. *avium* reference genome DSM 44156 (accession number: CP046507.1). Highlighted samples correspond to the sequence cluster calculated with SNV threshold from SNV pairwise distance upper boundary within patient diversity. The scale bar shows the number of SNVs per site (434.75 SNVs), and node bootstrap scores are shown when below 75 (with 1,000 bootstrap). The tree figure was annotated with additional information in columns: Patient, from which the strain was isolated; rPinecone, cluster number generated from rPinecone R analysis; SKA cluster, cluster number generated from SKA analysis; and ST, sequence type profile number obtained from the pubMLST *Mycobacteria* spp. scheme.

To study the genetic relationship of SGM causing chronic pulmonary disease, the sequences of all isolates from the same species were separated, and the sequence clusters obtained in different subtrees were analyzed. Different phylogenetic trees using the closest related genome to the sequence cluster as a reference for mapping reads against were inferred (Figures 2–4).

Subtree sequence cluster analysis using the SNV threshold methodology shows eight sequence clusters. Clusters II, IV, V, VI, VII, and VIII are formed by sequences from isolates from the same patient. Cluster I is formed by two *M. intracellulare* subsp. *chimaera* isolates from two different patients. Cluster III is formed by two *M. intracellulare* subsp. *intracellulare* isolates from two different patients.

Our study found that NTM isolates from different species and subspecies from the same complex have different SNVs and different genetic characteristics. When subclustering the isolates, every subtree showed sequence clusters formed by isolates from different patients with no apparent epidemiological relationship. In this study, all *M. intracellulare* subsp. *chimaera* isolates that cause chronic pulmonary disease were found to possess the same sequence type by MLST profiling, with the exception of one isolate (BCN_22_1), which exhibited a single difference compared to the others. Cluster I, supported by rPinecone analysis, SKA *kmer* clustering, and the SNV threshold (<1 SNV), is formed by two isolates from two different patients. Isolate BCN_01 from patient 1 and isolate BCN_33 from patient 33 were isolated from samples collected in the same hospital department but with a time difference greater than 1 year (419 days). No epidemiological relationship was found between these two patients.

Three sequence clusters were found in *M. intracellulare* subsp. *intracellulare* isolates. The SNV threshold methodology showed two clusters (Cluster IV and Cluster V) formed by two isolates from the same patient. Cluster III was formed by two very closely related isolates BCN_32_2 and BCN_38. Both isolates were from different patients with no demonstrated epidemiological relationship. BCN_32_2 was isolated from a sample received from another hospital, while BCN_38 was isolated from the Infectious Diseases Department of the Hospital Clinic of Barcelona. There was a time period of more than 3 years between the two isolates.

In Cluster IV, the SNV threshold approach clustered BCN_10_1 and BCN_10_2 together, but not the BCN_47 strain. The SNV difference between BCN_10_1 and BCN_10_2 is 0 SNVs while both strains differed from BCN_47 in 4 SNVs. This result shows a very close relationship between strains from different patients with no epidemiological relationship. The SKA methodology and the rPinecone analysis also showed that these three samples were very closely related.

## 4 Discussion

The most important findings of this study are as follows: First, the use of three methods of analysis is a useful strategy to epidemiologically classify clinical isolates of MAC. The most accurate approach to a good NTM classification is Roary core-genome tree analysis with type strain reference genomes. SNV threshold calculation and the SKA tool are good methodologies for studying the phylogenetic relationship between very closely related isolates from patients with bronchiectasis. Second, among the 46 isolates analyzed, sequence clustering showed a very close genetic relationship in 10 *M. intracellulare* strains. These strains were divided into five clusters. Three clusters consisted of two different isolates from the same patient (Clusters II, IV and V, from patients 19, 10, and 26, respectively). The other two clusters consisted of two different isolates from different patients (Cluster I, formed by isolates from patients 1 and 33, and Cluster III, formed by isolates from patients 32 and 38).

The Roary core-genome maximum likelihood tree, which included NTM first isolates and 12 reference strain genomes, revealed that strain BCN_21 was closer to the genome of the *M. intracellulare* MIP (*indicus pranii*) type strain. Although the *M. intracellulare* MIP strain is classified as *M. intracellulare* subsp. *intracellulare*, our findings suggest that it may be closer to the *M. paraintracellulare* species. This is in agreement with the findings by Castejon et al. (2018), who showed that MIP was closer to *M. intracellulare* MOTT64, which is the *M. paraintracellulare* type strain according to LPSN (https://lpsn.dsmz.de/) (Castejon et al., 2018; Nouioui et al., 2018).

The use of three different methodologies to cluster the isolates is important due to the differences in the clustering algorithms. On one hand, the SNV threshold analysis is a good approach to define highly related isolates based on empirical intrapatient SNV diversity when data from different isolates from the same patient is available (Doyle et al., 2020). On the other hand, the rPinecone methodology allows hierarchical clustering while maintaining a phylogenetic context (Wailan et al., 2019). The Split K-mer Analysis is particularly useful for surveillance studies as it detects highly related isolates without requiring full genome alignment (Harris, 2018). These methods complement each other and provide a robust framework for assessing genetic relatedness.

Sequence clustering showed that some *M. intracellulare* strains from non-epidemiologically related patients were very closely related at the core-genome level. To our knowledge that has been proven for other species, especially for rapidly growing mycobacteria such as *M. abscessus* complex, but not for *M. intracellulare* subspecies (Ruis et al., 2021). The SNV threshold sequence cluster analysis of *M. avium* subsp. *avium* isolates revealed 3 clusters. Clusters VI, VII, and VIII correspond to isolates from the same patients. This result agrees with Mizzi et al. (2022) who suggested that *M. avium* subsp. *avium* type *hominissuis* has the smallest core genome and the largest accessory genome. This indicates the higher level of diversity within the isolates belonging to this subspecies (Uchiya et al., 2017).

The results of rPinecone and SKA were similar to the SNV threshold method with a single difference; *M. intracellulare* subsp. *intracellulare* Cluster IV, clustered by the SNV threshold method and the rPinecone method, were able to separate isolates from the same patient from others, while SKA clustered 2 isolates from patient 10 with an isolate from patient 47. All three methods showed the presence of closely related isolates among patients but were not supported by epidemiological data. The use of a custom SNV threshold calculated in every analysis is important to study very closely related isolates, especially in bacterial populations not so deeply studied. The use of a master threshold number when calculating differences may not be suitable for all species, subspecies, or even lineages of bacteria. The evolutionary pressure and the adaptation mechanisms of the genome in every NTM species, subspecies or lineages may not be the same. A clear example is *M. tuberculosis*, which has a very conservative genome, which makes it easier to establish a unique SNV threshold (Srilohasin et al., 2020). Nevertheless, other NTM, such as *M. abscessus* or *M. avium* complex can modify their genomes by horizontal gene transfer and have other mutation rates (Kannan et al., 2019; Choo et al., 2014; Sapriel et al., 2016). The SNV threshold proved to be an effective approach, but it must be calculated for every analysis, when possible. The SKA methodology performed exceptionally well in our data and proved to be quicker than the other two analyses. The most notable advantage of SKA is that it can be applied directly to the trimmed reads. As previous studies suggest, NTM are widely present in natural and human-made environments, including water systems, soil, and biofilms in hospital settings (6, 8). These environmental reservoirs likely contribute to the observed genomic clustering among unrelated patients.

The strengths of this study include the sequencing of NTM pulmonary isolates from the same complex but of different species and subspecies, as well as the use and comparison of three different methodologies to generate and analyze sequence clustering for very closely related isolates. The finding of clusters from isolates from the same patient obtained over a period of 8–27 months reinforces the discriminatory power of the methodology applied. Moreover, the study brings some light to the phylogeny of *M. avium* complex isolates as a whole. This study also shows the differences between core-genome phylogenetic sequence cluster analysis and SNV threshold calculation for 3 different NTM subspecies and how each behaves differently when clustering. We found that isolates of *M. intracellulare* subsp. *chimaera* from our area are more closely related in terms of core-genome phylogeny than those of *M. avium* subsp. *avium*.

The main limitation of this study is the low number of isolates from the same subspecies and the unequal distribution of isolates for each subspecies. Although all the samples studied were from patients with bronchiectasis, they were obtained from routine laboratory work. In the period duration of this study, we were not able to find more isolates which met the inclusion criteria. Future work is required to increase the number of samples. Also, it would be interesting to include mycobacteria isolates that caused other types of infections, such as soft tissue infections or bacteraemia. This inclusion of different type of isolates would be an additional value to understand if the clustering here present is also present with isolates not causing bronchiectasis. Another limitation of the study is that the lack of complete patient histories limits the ability to determine shared environmental exposure definitively. Furthermore, our study lacks information on how variations in sample collection methods could influence the interpretation of clustering results. Future studies incorporating epidemiological data and environmental sampling will be essential to strengthen our conclusions. Finally, although there is no direct evidence for specific environmental sources of acquisition, our genomic findings strongly suggest this type of acquisition. Studies such as Hoefsloot et al. (2013) and Zweijpfenning et al. (2018) highlight the widespread presence of NTMs in water systems and reinforce the plausibility of the hypothesis of this study.

In conclusion, this study demonstrates that using the three methodologies of analysis is an adequate strategy for an in-depth study of the relationship between isolates belonging to closelt related species and subspecies. The results indicate the presence of very closely related isolates from different patients without any established epidemiological relationship. This finding suggests that the probable acquisition of infection from common sources is more than direct transmission between the patients. Furthermore, this study shows the low genomic diversity of *M. intracellulare* subspecies alongside a higher level of diversity among the *M. avium* isolates.

## Data Availability

The datasets presented in this study can be found in online repositories. The names of the repository/repositories and accession number(s) can be found here: https://www.ncbi.nlm.nih.gov/, PRJNA778929.

## Footnotes

^1^ https://pubmlst.org/bigsdb?db=pubmlst_mycobacteria_seqdef
